# Acetabular coverage exerts minimal influence on femoral head collapse and the necessity for surgical intervention in patients with osteonecrosis of femoral head

**DOI:** 10.1007/s00264-024-06238-w

**Published:** 2024-06-20

**Authors:** Yasuaki Kuriyama, Hidetatsu Tanaka, Kazuyoshi Baba, Ryuichi Kanabuchi, Yu Mori, Toshimi Aizawa

**Affiliations:** Department of Orthopaedic Surgery, Tohoku University Graduate School of Medicine, 1-1 Seiryo-machi, Aoba-ku, Sendai, 980-8574 Japan

**Keywords:** Osteonecrosis of the femoral head, Collapse of the femora head, Acetabular coverage, Surgical treatment, Natural course

## Abstract

**Purpose:**

The acetabular coverage in osteonecrosis of the femoral head (ONFH) affects the need for surgical intervention, and the collapse of the femoral head remains unclear. This study aimed to evaluate the relation between the acetabular coverage and the need for surgical treatment and femoral head collapse.

**Methods:**

The study included 158 patients with 252 hips with glucocorticoid administration and idiopathic ONHF without osteoarthritis changes. The mean age at the first visit was 45.2 years, and the mean follow-up period was 92.2 months. All ONFH hips were subsequently divided into two groups: those needing surgical intervention and those without surgery. Additionally, it divided 167 initially non-collapsed hips into those that either later collapsed or not. Radiographic parameters with the centre-edge angle, acetabular roof obliquity, sharp angle, and necrotic location, following the guidelines of the Japanese Investigation Committee, were evaluated.

**Results:**

There were no significant differences in radiographic parameters between the 106 hips that underwent surgery and the 146 hips without surgery. Among the 167 hips without initial collapse, 91 eventually collapsed while 76 did not; their radiographic findings have no significant differences. The necrotic locations were significantly larger in hips requiring surgical intervention or femoral head collapse. Furthermore, 21.8% (55 out of 252 hips) had acetabular dysplasia, which did not significantly correlate with the necessity for surgical treatment or the incidence of femoral head collapse.

**Conclusions:**

Acetabular coverage has little effect on the necessity for surgical treatment and femoral head collapse in ONFH patients over a long-term follow-up.

## Introduction

Osteonecrosis of the femoral head (ONFH) is a common hip disease that is thought to have a relationship with trauma, glucocorticoid use, alcohol abuse, autoimmune diseases, and other pathogenic factors [1–3]. ONFH impacts patients’ mobility and quality of life and is an increasing global health problem [4–6]. The national epidemiologic survey in 2004 estimated that for ONFH, approximately 11,400 patients in Japan were treated annually, and 2200 patients per year were newly diagnosed with this disease [7]. The prevalence of ONFH in males is higher than that of females; the peak decades of age in the final diagnosis were young, the 40s in male patients, and the 30s in females [7]. Femoral head collapse induces severe hip pain, resulting in surgical treatment such as total hip arthroplasty or osteotomy [8, 9].

The previous study reported predicting female head collapse factors: necrotic location, volume, location, and age of the head [10–12]. The progression of the femoral head collapse in patients with ONFH is associated with the size of necrosis location, volume [12–17]. A number of studies have demonstrated that acetabular anatomical abnormalities that caused incomplete contact surfaces between the acetabulum and the femoral head could increase intracapsular pressure [18–20]. Acetabular anatomical parameters were reported to affect femoral head collapse [21–23], a study showed lower acetabular coverage was associated with femoral head collapse in patients with ONFH [22], on the other hand, small association between lower acetabular coverage and higher odds were detected [21]. Lower acetabular coverage might be associated with the development of ONFH in East Asian populations [23]. Acetabular dysplasia was a significant factor associated with hip osteoarthritis (OA) in Japan [24]. Acetabular bone morphology is also likely to be involved in ONFH surgical treatment. Inadequate acetabular coverage increases joint contact pressure and may result in progressive femoral head collapse; rotational acetabular osteotomy was performed for ONFH [25].

The relationship between acetabular coverage and femoral head collapse in ONFH patients without initial collapse lacks consensus. Specifically, the connection between acetabular bone morphology and the need for surgical intervention remains uncertain. Therefore, this study aimed to assess whether (1) acetabular coverage in early ONFH without collapse predicts femoral head collapse, and (2) acetabular coverage is associated with the need for surgical treatment in ONFH patients over long-term follow-up.

## Materials and methods

### Participants

The institutional review board approved this longitudinal, retrospective study. This study was conducted in accordance with the ethical standards of the Declaration of Helsinki. We retrospectively analyzed 487 ONFH hip joints in 303 patients who visited our hospital from March 1979 to April 2024. All ONFH patients were diagnosed using clinical physical examination and imaging and met the Japanese Investigation Committee (JIC) diagnostic criteria [26], which require any two positive criteria out of the following five criteria: (1) collapse of the femoral head without joint-space narrowing or acetabular abnormality on x-rays (including crescent sign), (2) Demarcating sclerosis in the femoral head without joint space narrowing or acetabular abnormality, (3) “Cold in hot” on bone scans, (4) Low-intensity band on T1-weighted images (bandlike pattern), and (5) trabecular and marrow necrosis on histology. The inclusion criteria of the present study were set to (1) the follow-up periods were longer than 24 months, (2) the complete imaging data, and (3) the no osteoarthritis change at the first visit. Exclusion criteria were as follows: (1) the imaging data were incomplete during the follow-up or imaging data with poor quality, (2) alcoholic, traumatic ONFH, and radiation therapy, and (3) patients who had previous hip surgery on the first visit.

**Fig. 1:**
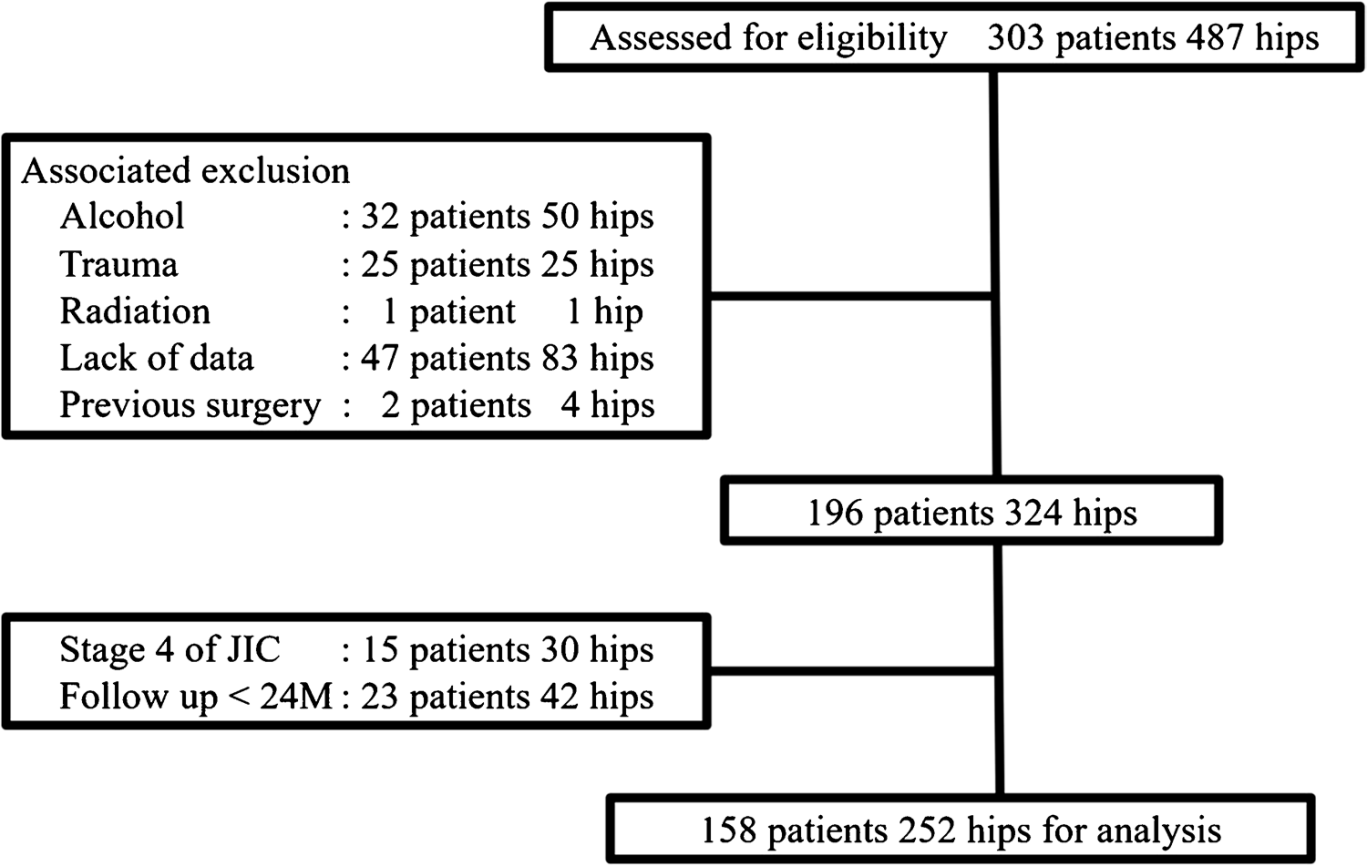
Flow diagram of patient selection

In this study, there were 158 patients (57 males and 101 females) totaling 252 hips, with 94 patients having bilateral ONFH. The mean age at the first visit was 45.2 years (14–79), and the mean follow-up period was 92.2 months (24–421). The etiology of ONFH was investigated; ONFH was associated with glucocorticoid administration in 94.8% (239 out of 252) and idiopathic in 5.1% (13 out of 252). The flow diagram of patient selection is shown in Fig. 1.

### Radiographic assessment

**Fig. 2:**
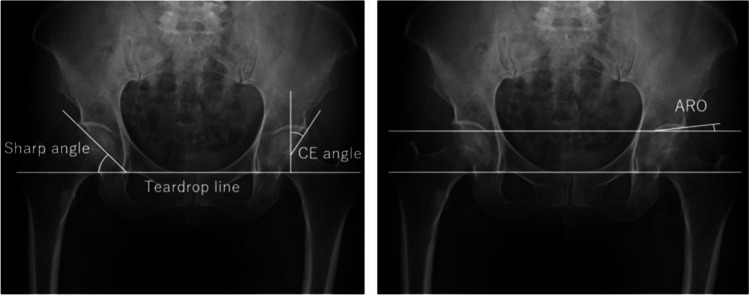
Anatomical parameters were evaluated, including center-edge (CE) angle, sharp angle, and acetabular roof obliquity (ARO). For the right hip, the angle measured from the line connecting the teardrops increases (positive value) in the clockwise direction and decreases (negative value) in the counter-clockwise direction

As a radiological evaluation for acetabular coverage, lateral center-edge (LCE) angle [27], acetabular roof obliquity (ARO) [28], and Sharp angle [29] at the simple anteroposterior radiographs in the supine position of the hip joint at the first visit (Fig. 2). Two orthopaedic surgeons (Y.K. and H.T.) measured for each parameter. Patients were followed up regularly, and anteroposterior radiographs of the hip were obtained in each follow-up to evaluate the collapse of the femoral head. The necrotic location of the weight-bearing area was classified into four types using the JIC classification (A, B, C1, and C2) [26]. ONFH progression at the first visit was evaluated according to JIC classification (stages 1, 2, 3 A, 3B, and 4) [26], while stage 4 was an unmet classification criterion. Whether the femoral head was collapsed or not was also evaluated. JIC classification scheme has been widely used in Japan and has high inter-observer and intra-observer reliability [30]. Acetabular dysplasia was defined as the presence of the following findings on AP pelvic radiographs: CE angle < 20°, ARO > 15°, or sharp angle > 45° [31]. All ONFH hips were subsequently divided into two groups, those with the need for surgical intervention and those who did not. The 167 ONFH hips without femoral collapse were also divided into two groups, those with femoral head collapse or not. We investigated whether the acetabular coverage affects the need for surgical intervention in all ONFH and whether the femoral head collapses in ONFH without the collapse of the femoral head. Whether the femoral head collapsed or not was decided by comparing the previous radiographic image. We also consider the acetabular dysplasia associated with the need for surgical intervention and femoral head collapse as a secondary evaluation.

### Statistical analysis

Statistical analysis was performed using JMP 17 (SAS Institute Japan Ltd., Tokyo, Japan). All quantitative data are presented as mean ± standard deviation. To compare the results of two independent groups, Student’s t-test and χ2 were used. To assess the reliability of the radiographic parameters, the intraclass correlation coefficient (ICC) is used to evaluate intra- and inter-observer. The comparison of two distinct results was conducted using an independent samples t-test. The Kaplan-Meier survival analysis was performed with the femoral head collapse for stages 1 and 2 and the operation for all stages as an endpoint between the dysplasia and non-dysplasia case. P-value < 0.05 was statistically significant.

## Results

At the latest follow-up, surgical intervention was performed on 106 hips (surgery group) with eight bipolar hip arthroplasty, 97 total hip arthroplasty, and one resection arthroplasty. In contrast, the remaining 146 hips did not undergo surgical treatment (non-surgery group). The necrotic location was significantly spread in surgical groups, and the ONFN phase was advanced (Table 1). The radiographic parameters of acetabular have no significant differences between the groups. The intra-and inter-observer reliability was almost fit, with an ICC value of 0.961 (95% Confidence Interval [CI]: 0.939 − 0.986) and 0.923 (95% CI: 0.892 − 0.946).

**Table 1:** Comparison of the JIC-tye, JIC-stage, and acetabular coverage between the surgical and non-surgical groups

	Surgical group	Non-surgical group	*p*-value
n	106	146	
Age	43.7 ± 14.0	46.2 ± 16.2	0.131
Gender n(%)			
Men	73 (45.6)	33 (35.9)	
Women	87 (54.4)	59 (64.1)	0.146
Etiology n(%)			
Steroid	99 (93.4)	140 (95.9)	
Idiopathic	7 (6.6)	6 (4.1)	0.377
JIC-Type n(%)			
A	1(0.94)	9(6.2)	
B	6(5.66)	20(13.7)	
C1	18(17.0)	48(32.9)	
C2	81(76.4)	69(47.3)	0.000*
JIC-Stage n(%)			
1	31(29.3)	81(55.5)	
2	18(17.0)	37(25.3)	
3A	40(37.7)	21(14.4)	
3B	17(16.0)	7(4.8)	0.000*
Radiographic paramters of acetanbular			
CE angle	29.9 ± 7.3	29.7 ± 6.5	0.798
ARO	7.2 ± 5.6	6.5 ± 5.9	0.353
Sharp angle	41.6 ± 4.0	41.0 ± 3.9	0.318

Age and radiographic paramters are shown as mean ± standard deviation;

Paired t-test was used to compare the age and radiographic paramters

χ2 test was used to compare the gender, etiology, JIC-type, and JIC- Stage

^*^ *p* < 0.05

The 167 hips were classified JIC-stage 1 or 2 at the first visit, ONFH without collapse. At the latest follow-up, 91 hips collapsed, while the remaining 76 hips did not collapse. In the collapsed hips, the type C2 ratio was significantly high, and the radiographic parameters of acetabular have no significant differences (Table 2).

**Table 2:** Comparison of the JIC-tye, JIC-stage, and acetabular coverage between the collapse and non-collapse groups

	Collapse	Non-collapse	*p*-value
n	91	76	
JIC-Type n(%)			
A	2 (2.2)	8 (10.5)	
B	9(9.9)	15 (19.7)	
C1	16 (17.6)	32 (42.1)	
C2	64 (70.3)	21 (27.6)	0.000*
Stage			
1	56 (61.5)	56 (73.7)	
2	35 (38.5)	20 (26.3)	0.095
Radiographic paramters			
CE angle	29.1 ± 6.5	29.1 ± 6.5	0.963
ARO	6.5 ± 5.4	7.1 ± 5.9	0.452
Sharp	41.4 ± 3.9	41.7 ± 3.8	0.712

Radiographic paramters is shown as mean ± standard deviation;

Paired t-test was used to compare theradiographic paramters

χ2 test was used to compare the gender, etiology, JIC-type, and JIC- Stage

^*^ *p* < 0.05

In addition, acetabular hip dysplasia was found in 55 of 252 hips (21.8%) of the ONFH hips. The surgical intervention rate at the latest follow-up showed no significant differences. However, the necrotic location and ONFN stage showed no significant differences between the dysplasia and non-dysplasia hips (Table 3). Acetabular dysplasia of the hip was found in 42 out of 167 hips (25.1%) of the hip with non-collapsed ONFH, and the femoral head collapse rate has no significant differences between the dysplasia and non-dysplasia group (Table 4).

**Table 3:** Comparison of the JIC-tye, JIC-stage, and acetabular coverage between the dysplasia and non-dysplasia hips

	Dysplasia	Non-dysplasia	*p*-value
n	55	197	
Surgical intervention at the latest follow up n(%)	25(45.5)	81 (41.1)	0.565
JIC-Type n(%)			
A	4 (7.3)	6 (3.0)	
B	8 (14.5)	18 (9.1)	
C1	14 (25.5)	52 (26.4)	
C2	29 (52.7)	121 (61.4)	0.338
Stage			
1	28 (50.9)	84 (50.9)	
2	14 (25.5)	41 (20.8)	
3A	9 (16.4)	52 (26.4)	
3B	4 (7.3)	20 (10.2)	0.333
Radiographic paramters			
CE angle	22.0 ± 0.7	31.9 ± 0.4	0.000*
ARO	12.6 ± 0.7	5.1 ± 0.3	0.000*
Sharp	46.0 ± 0.4	40.0 ± 0.2	0.000*

Radiographic paramters is shown as mean ± standard deviation;

Paired t-test was used to compare theradiographic paramters

χ2 test was used to compare the operation rate at the latest follow up, JIC-type, and JIC- Stage

^*^ *p* < 0.05

**Table 4:** Comparison of the JIC-tye, JIC-stage, and acetabular coverage between the dysplasia and non-dysplasia hips of ONFH without collapse

	Dysplasia	Non-dysplasia	*p*-value
n	42	125	
Femoral head collapse at the latest follow up n(%)	23 (54.8)	68 (54.4)	0.968
JIC-Type n(%)			
A	4(9.5)	6 (4.8)	
B	8(19.1)	16(12.8)	
C1	12(28.6)	36(28.8)	
C2	18(42.9)	67(53.6)	0.457
Stage			
1	28(66.7)	84(67.2)	
2	14(33.3)	41(32.8)	0.949
Radiographic paramters			
CE angle	22.2 ± 0.8	31.5 ± 0.4	0.000*
ARO	12.0 ± 0.7	5.0 ± 0.4	0.000*
Sharp	46.0 ± 0.4	40.1 ± 0	0.000*

Radiographic paramters is shown as mean ± standard deviation;

Paired t-test was used to compare theradiographic paramters

χ2 test was used to compare the femoral head collapse rate, JIC-type, and JIC- Stage

^*^ *p* < 0.05

**Fig. 3:**
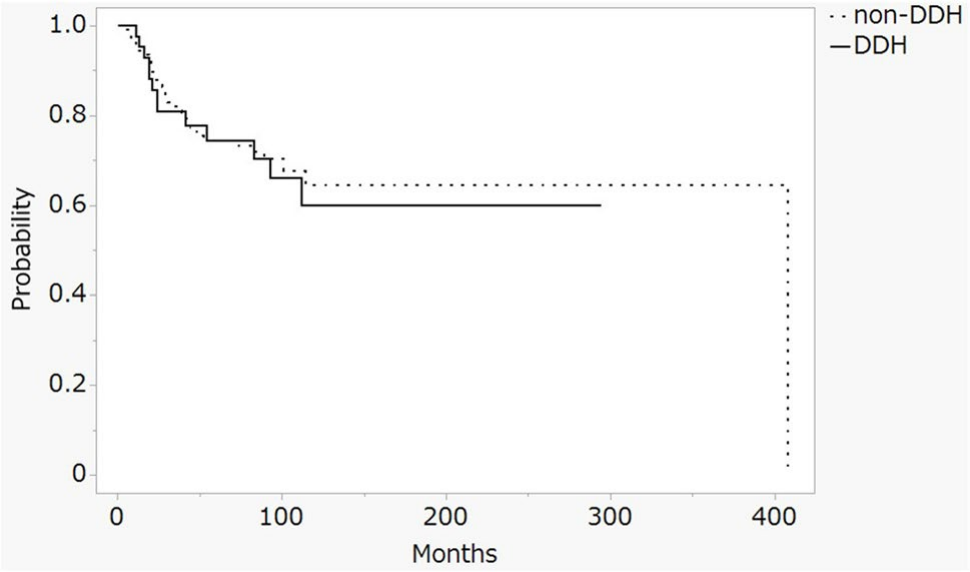
Kaplan–Meier survivorship analysis at 10 years with the operation shows 60.0% in the dysplasia hip (95% CI 41.1–78.8) and 64.6% in the non-dysplasia hip (95% CI 53.4–75.9). DDH: developmental dysplasia of the hip

**Fig. 4:**
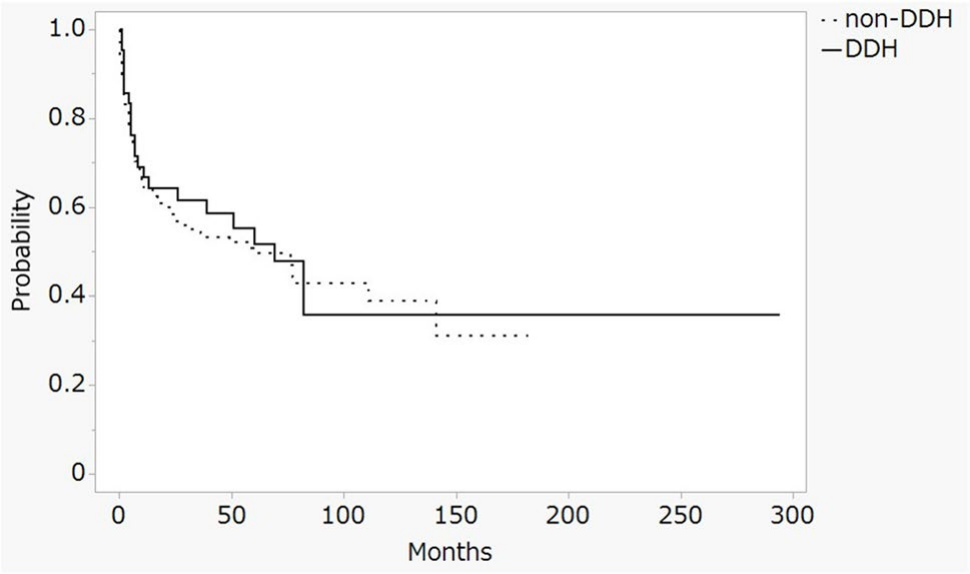
Kaplan–Meier survivorship analysis at 10 years with the collapse of the femoral head as the 38.6% in dysplasia hip (95% CI 29.2–47.7) and 39.0% in the non-dysplasia hip (95% CI 27.7–50.3). DDH: developmental dysplasia of the hip

At the ten-year evaluation point, Kaplan-Meier survival analysis revealed no statistically significant differences in survival rates between the two groups when using surgical intervention as the endpoint. Specifically, the survival rate for dysplastic hips was 60.0% (95% CI: 41.1–78.8), compared to 64.6% (95% CI: 53.4–75.9) for non-dysplastic hips, with a p-value of 0.720 (Fig. 3). Additionally, in cases of non-collapsed ONFH, with femoral head collapse as the outcome, the survival rates were 38.6% (95% CI: 29.2–47.7) for dysplastic hips and 39.0% (95% CI: 27.7–50.3) for non-dysplastic hips, showing no statistically significant difference between the groups (p-value of 0.939) (Fig. 4).

## Discussion

The study involved 158 patients with 252 hips with glucocorticoid-induced and idiopathic ONFH. Our data shows acetabular coverage appears to have little association with the collapse in patients with non-collapsed ONFH and surgical treatment in patients with ONFH without osteoarthritis change. Additionally, the incidences of acetabular dysplasia were 55 hips (21.8%), and acetabular coverage has little association with the collapse in patients with non-collapsed ONFH and surgical treatment in patients with ONFH. The ten-year survival rate from the first visit shows no significant differences between dysplasia cases and non-dysplasia hips.

Previous studies have reported the relationship between acetabular morphologies and femoral head collapse. Tianye Lin et al. evaluated 135 patients with 151 hips with non-traumatic ONFH. They showed that the hip joint medial space ratio (MSR) and CE angle strongly correlate with collapse, except for assessing the relationship between the femoral head and the acetabulum [22]. MSR is an indication of external femoral head movement and has high specificity. When MSR is > 20.35, the rate of collapse of ONFH will increase drastically [22]. Junfeng Zeng et al. compared the radiographic hip pathologies between 101 patients with 136 hips with idiopathic ONFH and 202 control subjects with 404 hips matched for age, gender, and body mass index who had no apparent radiographic hip pathologies. The idiopathic ONFH patients had less acetabular coverage, lower CE angle, acetabular depth ratio, acetabular head index, and higher sharp angle than the control subjects with no apparent hip osseous abnormalities [23]. They concluded that less acetabular coverage was found in hips with idiopathic osteonecrosis than in control subjects [23]. On the other hand, Isawa et al. thoroughly investigated 343 hips in 218 patients with ONFH using computed tomography (CT) images. They demonstrated that acetabular coverage appears to have little association with the likelihood of collapse in patients with ONFH, with a small association between a lower lateral CE angle and a higher odd of collapse. Still, the effect size may not be clinically meaningful [21]. Hernigou et al. showed abnormal spino-pelvic, especially high pelvic incidence (PI) could affect the progression of ONFH and aggravate the risk of collapse [32, 33]. As corticosteroid is a cause of disc degeneration, the spine posture and spino-pelvic parameters may be changed in ONFH related to corticosteroid, and therefore the relative location of osteonecrosis to acetabulum may be changed [32, 33]. Conversely, Isawa et al. measured PI using CT images, and PI was not associated with femoral head collapse [21]. In the present study, for a long-term follow-up of 252 ONFH hips, the acetabular coverage has no significant correlation with the collapse and surgical treatment in patients with ONFH, even the hips with acetabular dysplasia.

The natural course of early ONFH without collapse was investigated, with the rate of collapse increasing with the width of the necrotic region and decreasing with the narrowing of the necrotic region [10–12]. A systematic review of untreated ONFH demonstrated natural histories of asymptomatic ONFH involve a high (84%) risk of progression of large lesions and a substantial (25%) risk of progression of medium-sized lesions (10). A progression of femoral head collapse in ONFH patients is associated with factors of necrotic area size, volume, and location [12–17]. The systematic review found that types A, B, and C of hip collapses occur in 9%, 19%, and 59% of ONFH patients, respectively [10]. In addition, cessation of collapse and improvement of symptoms without surgical intervention can occur in patients with type A and B hips once the femoral head has collapsed [7, 12, 34]. Most of the type-A hips survived without collapsing and progressive symptoms. Therefore, type A and type B hips with no or mild symptoms would allow to be observed without treatment [7]. The C-type group has shown poor prognosis (C1 and C2). In other words, it is a case where femoral head collapse was initiated [7]. In the present study, the prominent necrosis location at the time of initial examination was a risk factor for requiring surgical treatment and femoral head collapse. The advanced stage at the initial examination was also a risk factor for requiring surgical treatment. These findings were consistent with the previous reports.

This study has certain limitations. The first limitation is that this study is a retrospective case-control study, and selection bias cannot be avoided. Most patients are diagnosed at consultation, not at the onset of symptoms. The second limitation is that the acetabular anatomical parameters were evaluated using anteroposterior hip radiographs. CT has been desirable for assessing bone morphology due to its accuracy [21]. Since the images were not acquired in all cases, the evaluation using CT images was abandoned. The third limitation is that the quantitative measurement of femoral head collapse has not been assessed. The indication for surgery may vary depending on the femoral head’s collapse and symptoms. In patients with type B ONFH, femoral head collapse stops, and the patient becomes asymptomatic and collapses to less than two mm [12]. The fourth limitation is the small number of patients and the single-center study. A larger, prospective, multi-center cohort study was desirable to confirm our findings.

## Conclusion

The acetabular coverage little impacts femoral head collapse in ONFH patients without collapse and surgical treatment in ONFH patients without OA change for long-term follow-up.

## Abbreviation

ONFH: Osteonecrosis of the Femoral Head
